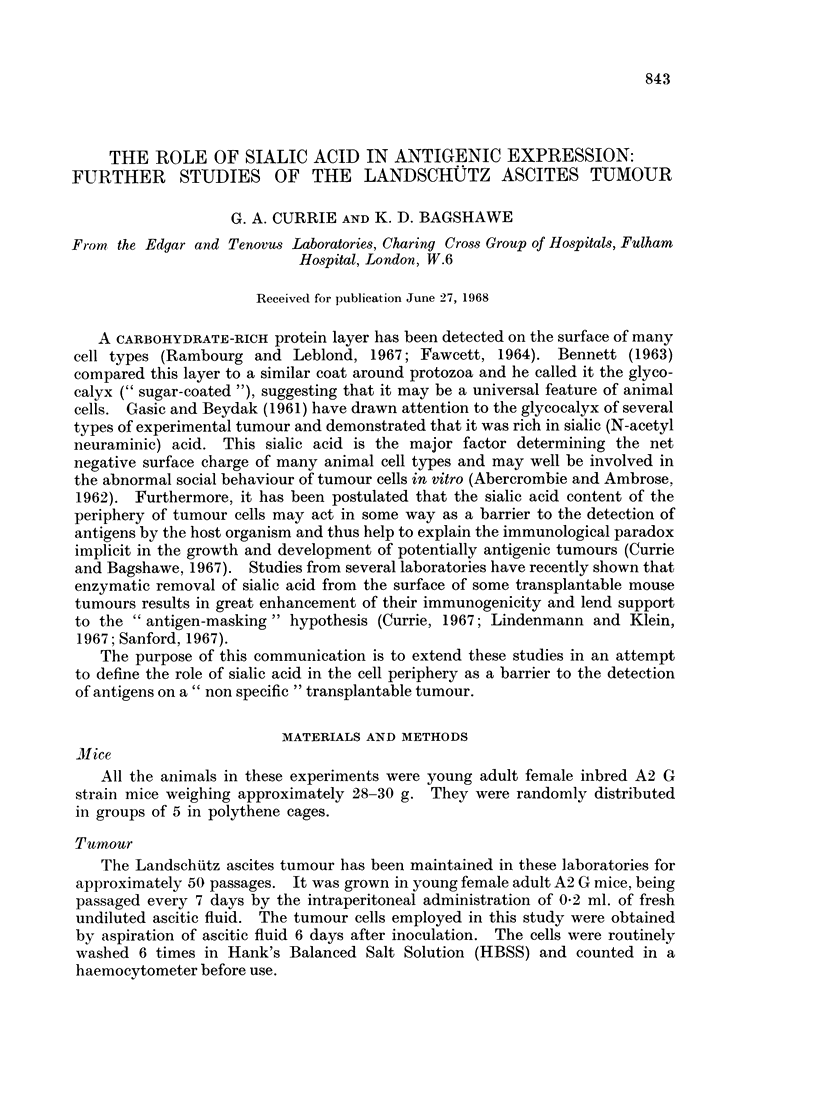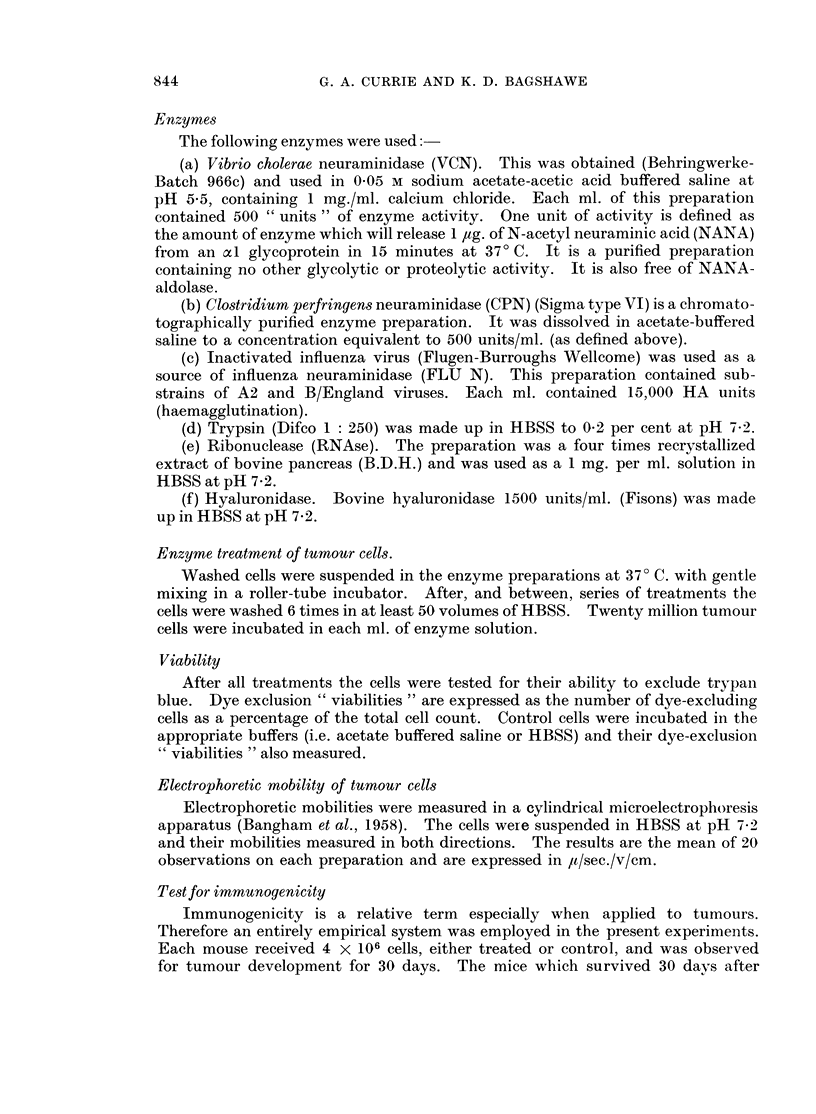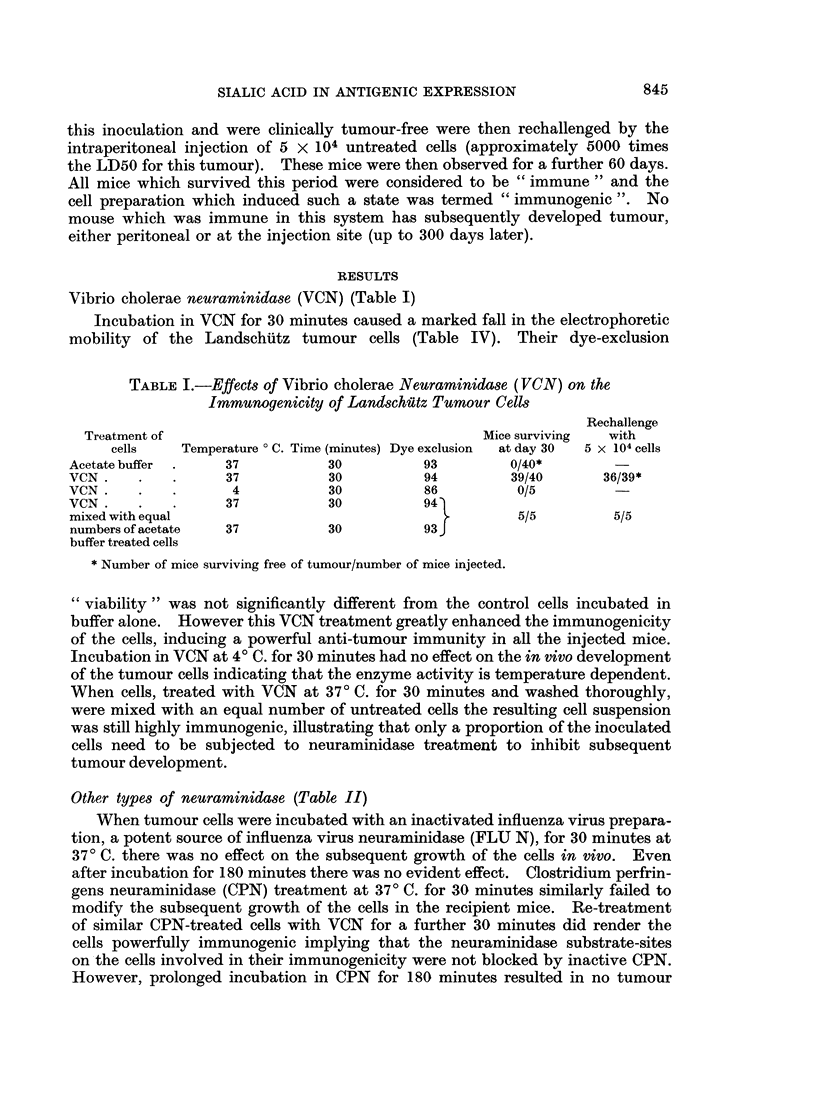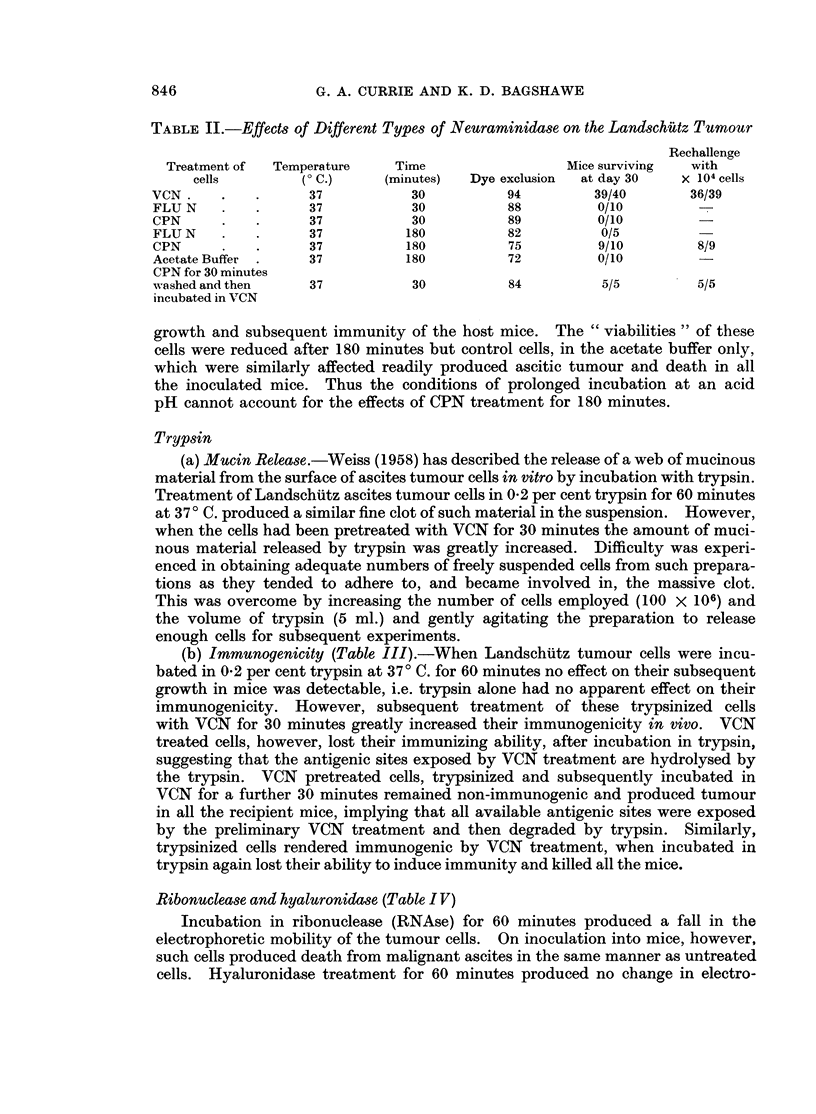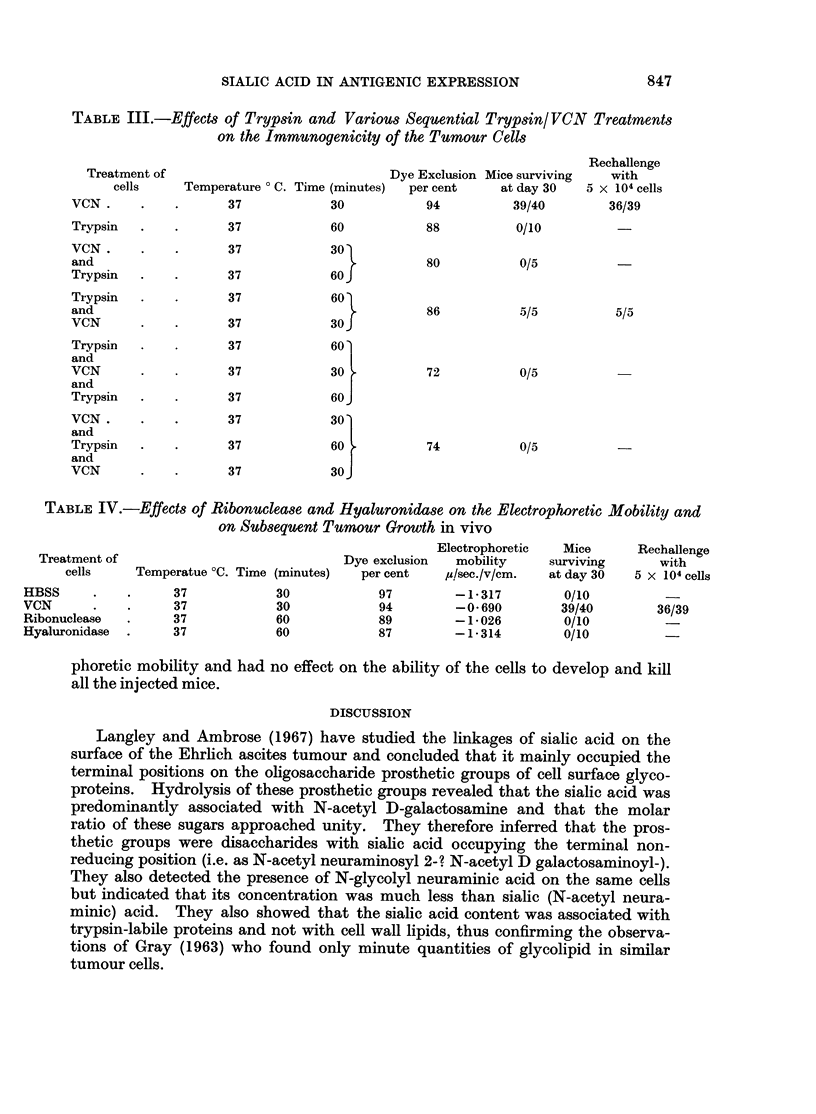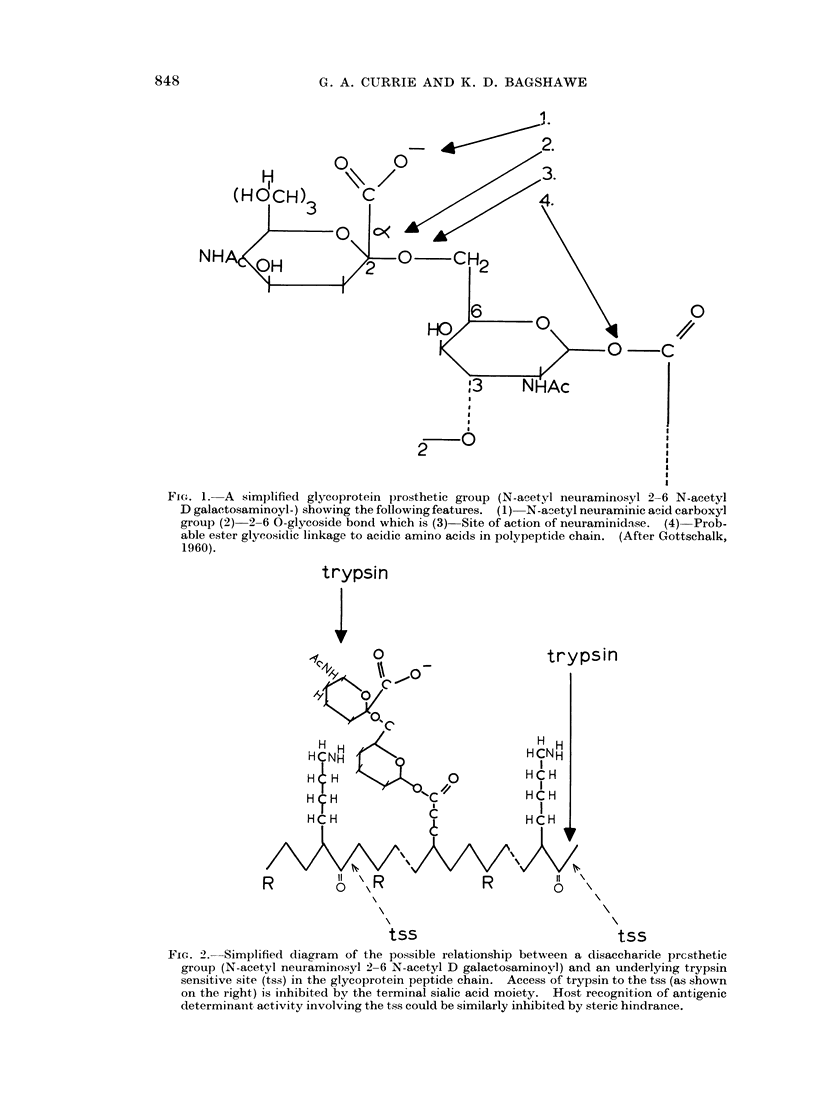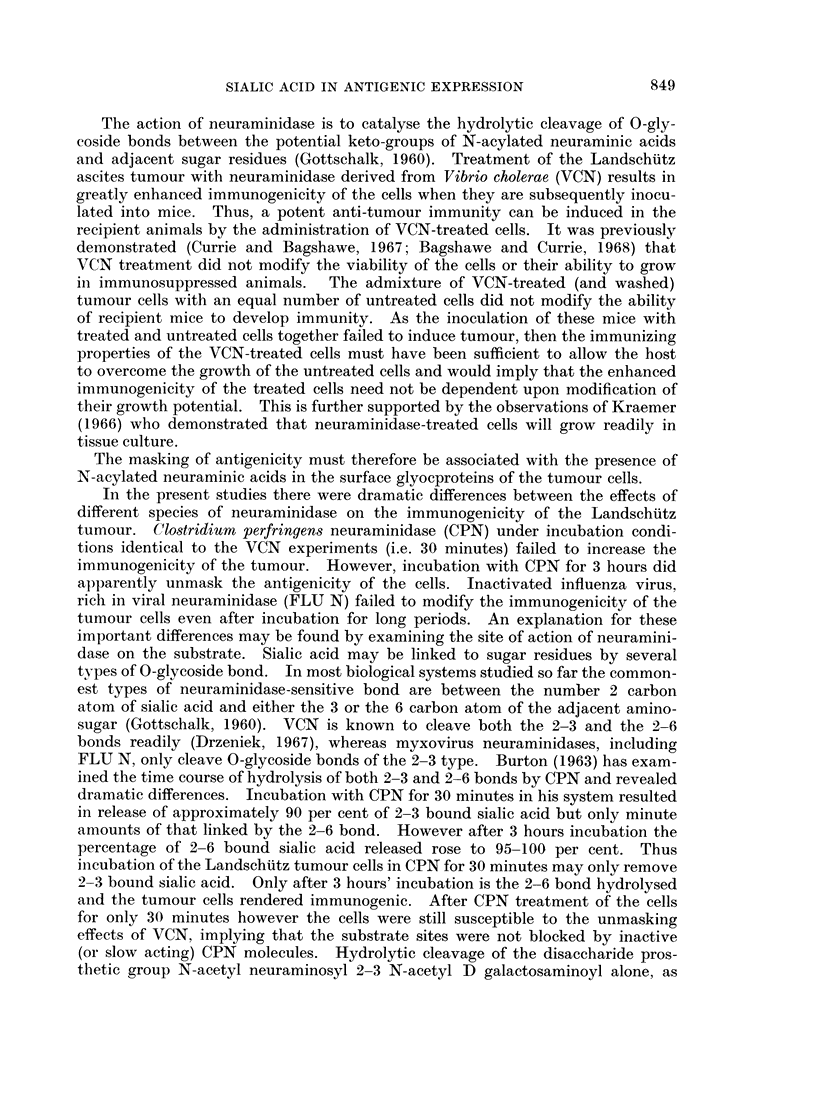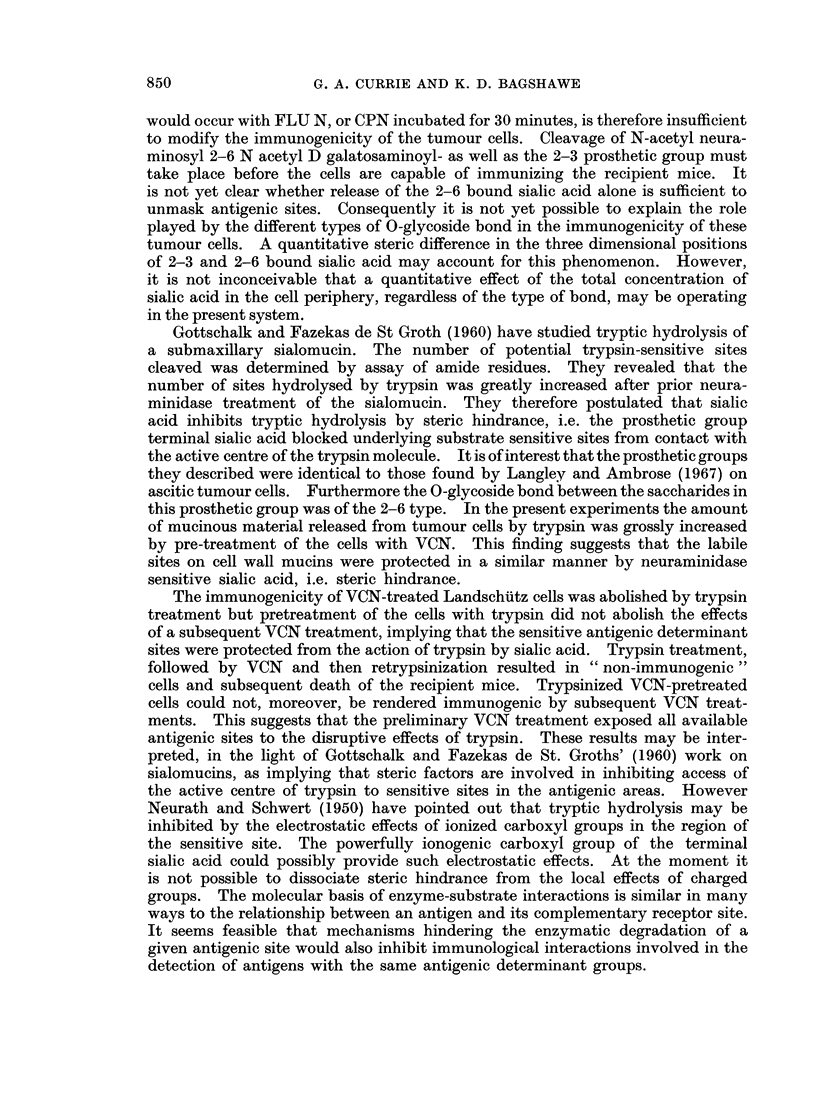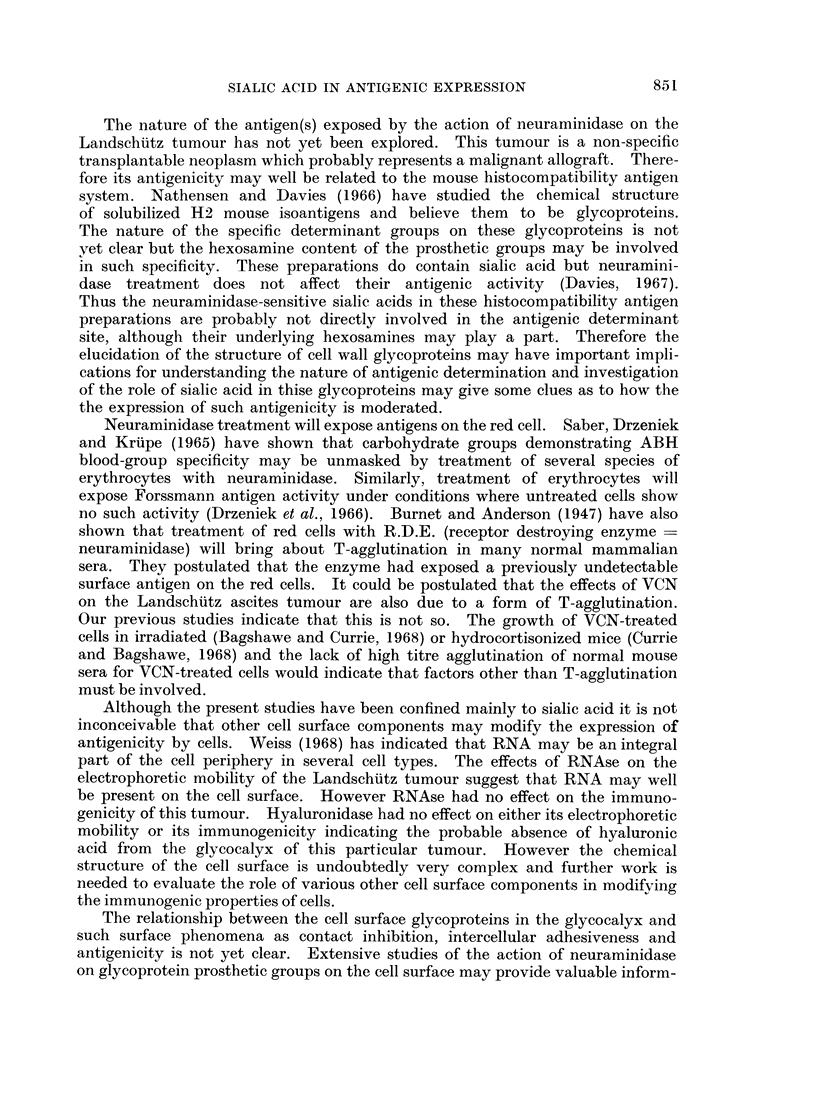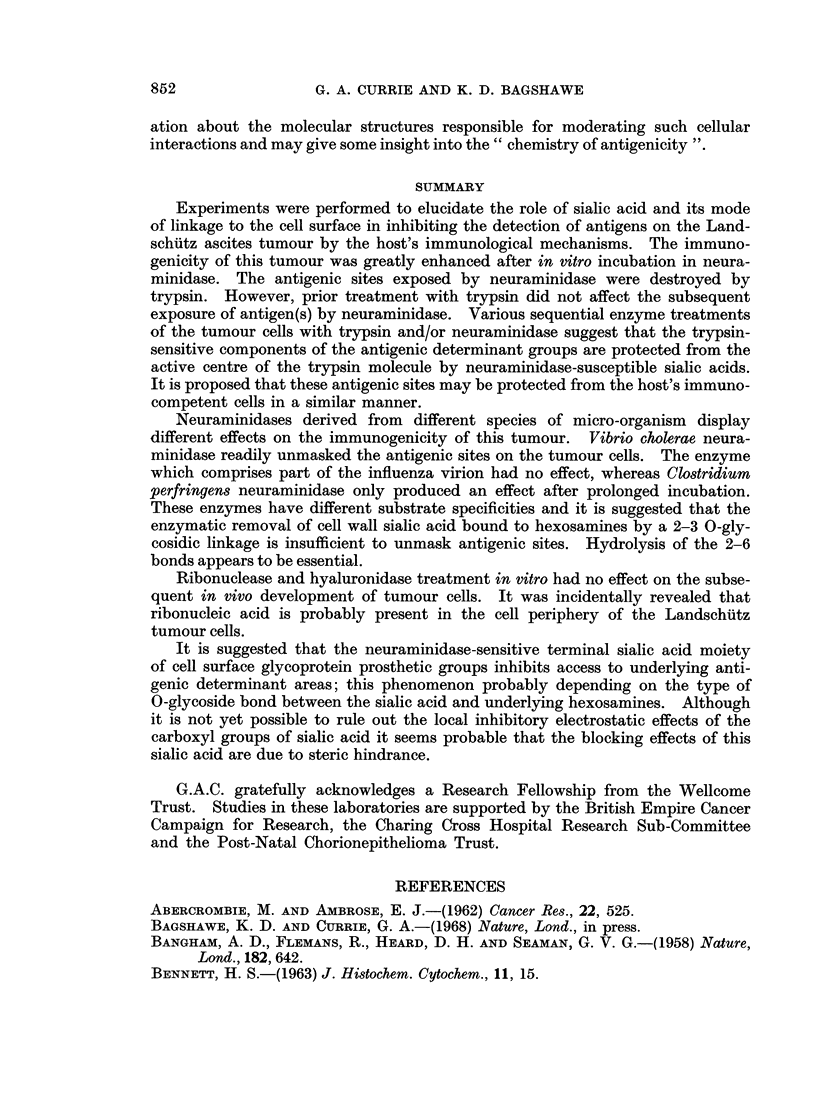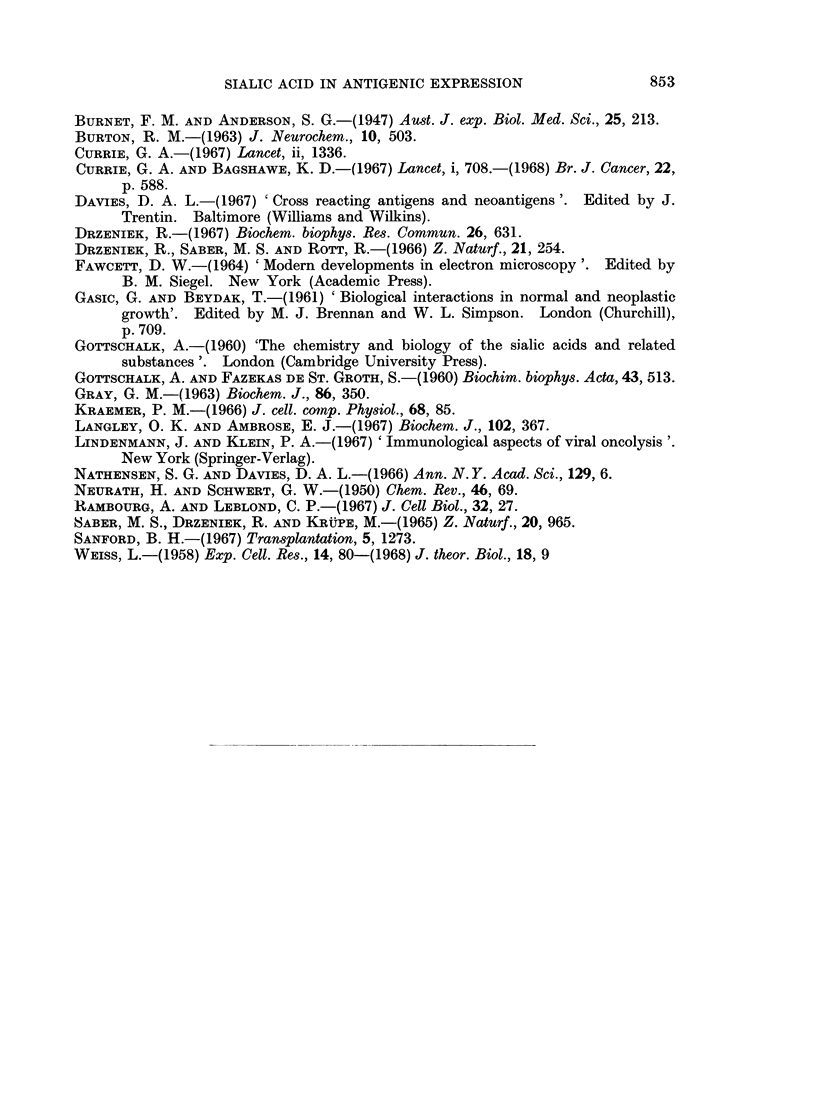# The role of sialic acid in antigenic expression: further studies of the Landschütz ascites tumour.

**DOI:** 10.1038/bjc.1968.99

**Published:** 1968-12

**Authors:** G. A. Currie, K. D. Bagshawe


### THE ROLE OF SIALIC ACID IN ANTIGENIC EXPRESSION: FURTHER STUDIES OF THE LANDSCHÜTZ ASCITES TUMOUR

G. A. CURRIE AND K. D. BAGSHAWE

*From the Edgar and Tenovus Laboratories, Charing Cross Group of Hospitals, Fulham Hospital, London, W.6*

Received for publication June 27, 1968

A CARBOHYDRATE-RICH protein layer has been detected on the surface of many cell types (Rambourg and Leblond, 1967; Fawcett, 1964). Bennett (1963) compared this layer to a similar coat around protozoa and he called it the glycocalyx (" sugar-coated "), suggesting that it may be a universal feature of animal cells. Gasic and Beydak (1961) have drawn attention to the glycocalyx of several types of experimental tumour and demonstrated that it was rich in sialic (N-acetyl neuraminic) acid. This sialic acid is the major factor determining the net negative surface charge of many animal cell types and may well be involved in the abnormal social behaviour of tumour cells *in vitro* (Abercrombie and Ambrose, 1962). Furthermore, it has been postulated that the sialic acid content of the periphery of tumour cells may act in some way as a barrier to the detection of antigens by the host organism and thus help to explain the immunological paradox implicit in the growth and development of potentially antigenic tumours (Currie and Bagshawe, 1967). Studies from several laboratories have recently shown that enzymatic removal of sialic acid from the surface of some transplantable mouse tumours results in great enhancement of their immunogenicity and lend support to the " antigen-masking " hypothesis (Currie, 1967; Lindenmann and Klein, 1967; Sanford, 1967).

The purpose of this communication is to extend these studies in an attempt to define the role of sialic acid in the cell periphery as a barrier to the detection of antigens on a " non specific " transplantable tumour.

### MATERIALS AND METHODS

### *Mice*

All the animals in these experiments were young adult female inbred A2 G strain mice weighing approximately 28–30 g. They were randomly distributed in groups of 5 in polythene cages.

### *Tumour*

The Landschütz ascites tumour has been maintained in these laboratories for approximately 50 passages. It was grown in young female adult A2 G mice, being passaged every 7 days by the intraperitoneal administration of 0·2 ml. of fresh undiluted ascitic fluid. The tumour cells employed in this study were obtained by aspiration of ascitic fluid 6 days after inoculation. The cells were routinely washed 6 times in Hank's Balanced Salt Solution (HBSS) and counted in a haemocytometer before use.

*Enzymes*

The following enzymes were used:—

(a) *Vibrio cholerae* neuraminidase (VCN). This was obtained (Behringwerke-Batch 966c) and used in 0·05 M sodium acetate-acetic acid buffered saline at pH 5·5, containing 1 mg./ml. calcium chloride. Each ml. of this preparation contained 500 " units " of enzyme activity. One unit of activity is defined as the amount of enzyme which will release 1 $\mu$g. of N-acetyl neuraminic acid (NANA) from an $\alpha$1 glycoprotein in 15 minutes at 37° C. It is a purified preparation containing no other glycolytic or proteolytic activity. It is also free of NANA-aldolase.

(b) *Clostridium perfringens* neuraminidase (CPN) (Sigma type VI) is a chromatographically purified enzyme preparation. It was dissolved in acetate-buffered saline to a concentration equivalent to 500 units/ml. (as defined above).

(c) Inactivated influenza virus (Flugen-Burroughs Wellcome) was used as a source of influenza neuraminidase (FLU N). This preparation contained substrains of A2 and B/England viruses. Each ml. contained 15,000 HA units (haemagglutination).

(d) Trypsin (Difco 1 : 250) was made up in HBSS to 0·2 per cent at pH 7·2.

(e) Ribonuclease (RNAse). The preparation was a four times recrystallized extract of bovine pancreas (B.D.H.) and was used as a 1 mg. per ml. solution in HBSS at pH 7·2.

(f) Hyaluronidase. Bovine hyaluronidase 1500 units/ml. (Fisons) was made up in HBSS at pH 7·2.

*Enzyme treatment of tumour cells.*

Washed cells were suspended in the enzyme preparations at 37° C. with gentle mixing in a roller-tube incubator. After, and between, series of treatments the cells were washed 6 times in at least 50 volumes of HBSS. Twenty million tumour cells were incubated in each ml. of enzyme solution.

*Viability*

After all treatments the cells were tested for their ability to exclude trypan blue. Dye exclusion " viabilities " are expressed as the number of dye-excluding cells as a percentage of the total cell count. Control cells were incubated in the appropriate buffers (i.e. acetate buffered saline or HBSS) and their dye-exclusion " viabilities " also measured.

*Electrophoretic mobility of tumour cells*

Electrophoretic mobilities were measured in a cylindrical microelectrophoresis apparatus (Bangham *et al.*, 1958). The cells were suspended in HBSS at pH 7·2 and their mobilities measured in both directions. The results are the mean of 20 observations on each preparation and are expressed in $\mu$/sec./v/cm.

*Test for immunogenicity*

Immunogenicity is a relative term especially when applied to tumours. Therefore an entirely empirical system was employed in the present experiments. Each mouse received $4 \times 10^6$ cells, either treated or control, and was observed for tumour development for 30 days. The mice which survived 30 days after this inoculation and were clinically tumour-free were then rechallenged by the intraperitoneal injection of $5 \times 10^4$ untreated cells (approximately 5000 times the LD50 for this tumour). These mice were then observed for a further 60 days. All mice which survived this period were considered to be " immune " and the cell preparation which induced such a state was termed " immunogenic ". No mouse which was immune in this system has subsequently developed tumour, either peritoneal or at the injection site (up to 300 days later).

### RESULTS

### Vibrio cholerae *neuraminidase* (VCN) (Table I)

Incubation in VCN for 30 minutes caused a marked fall in the electrophoretic mobility of the Landschütz tumour cells (Table IV). Their dye-exclusion

TABLE I.—*Effects of* Vibrio cholerae *Neuraminidase* (*VCN*) *on the Immunogenicity of Landschütz Tumour Cells*

| Treatment of cells | Temperature ° C. | Time (minutes) | Dye exclusion | Mice surviving at day 30 | Rechallenge with $5 \times 10^4$ cells |
|---|---|---|---|---|---|
| Acetate buffer | 37 | 30 | 93 | 0/40* | — |
| VCN | 37 | 30 | 94 | 39/40 | 36/39* |
| VCN | 4 | 30 | 86 | 0/5 | — |
| VCN mixed with equal numbers of acetate buffer treated cells | 37 / 37 | 30 / 30 | 94 / 93 | 5/5 | 5/5 |

* Number of mice surviving free of tumour/number of mice injected.

" viability " was not significantly different from the control cells incubated in buffer alone. However this VCN treatment greatly enhanced the immunogenicity of the cells, inducing a powerful anti-tumour immunity in all the injected mice. Incubation in VCN at 4° C. for 30 minutes had no effect on the *in vivo* development of the tumour cells indicating that the enzyme activity is temperature dependent. When cells, treated with VCN at 37° C. for 30 minutes and washed thoroughly, were mixed with an equal number of untreated cells the resulting cell suspension was still highly immunogenic, illustrating that only a proportion of the inoculated cells need to be subjected to neuraminidase treatment to inhibit subsequent tumour development.

### *Other types of neuraminidase* (*Table II*)

When tumour cells were incubated with an inactivated influenza virus preparation, a potent source of influenza virus neuraminidase (FLU N), for 30 minutes at 37° C. there was no effect on the subsequent growth of the cells *in vivo*. Even after incubation for 180 minutes there was no evident effect. Clostridium perfringens neuraminidase (CPN) treatment at 37° C. for 30 minutes similarly failed to modify the subsequent growth of the cells in the recipient mice. Re-treatment of similar CPN-treated cells with VCN for a further 30 minutes did render the cells powerfully immunogenic implying that the neuraminidase substrate-sites on the cells involved in their immunogenicity were not blocked by inactive CPN. However, prolonged incubation in CPN for 180 minutes resulted in no tumour

TABLE II.—*Effects of Different Types of Neuraminidase on the Landschütz Tumour*

| Treatment of cells | Temperature (° C.) | Time (minutes) | Dye exclusion | Mice surviving at day 30 | Rechallenge with × $10^4$ cells |
|---|---|---|---|---|---|
| VCN . . . | 37 | 30 | 94 | 39/40 | 36/39 |
| FLU N . . | 37 | 30 | 88 | 0/10 | — |
| CPN . . | 37 | 30 | 89 | 0/10 | — |
| FLU N . . | 37 | 180 | 82 | 0/5 | — |
| CPN . . | 37 | 180 | 75 | 9/10 | 8/9 |
| Acetate Buffer . | 37 | 180 | 72 | 0/10 | — |
| CPN for 30 minutes washed and then incubated in VCN | 37 | 30 | 84 | 5/5 | 5/5 |

growth and subsequent immunity of the host mice. The " viabilities " of these cells were reduced after 180 minutes but control cells, in the acetate buffer only, which were similarly affected readily produced ascitic tumour and death in all the inoculated mice. Thus the conditions of prolonged incubation at an acid pH cannot account for the effects of CPN treatment for 180 minutes.

*Trypsin*

(a) *Mucin Release.*—Weiss (1958) has described the release of a web of mucinous material from the surface of ascites tumour cells *in vitro* by incubation with trypsin. Treatment of Landschütz ascites tumour cells in 0·2 per cent trypsin for 60 minutes at 37° C. produced a similar fine clot of such material in the suspension. However, when the cells had been pretreated with VCN for 30 minutes the amount of mucinous material released by trypsin was greatly increased. Difficulty was experienced in obtaining adequate numbers of freely suspended cells from such preparations as they tended to adhere to, and became involved in, the massive clot. This was overcome by increasing the number of cells employed (100 × $10^6$) and the volume of trypsin (5 ml.) and gently agitating the preparation to release enough cells for subsequent experiments.

(b) *Immunogenicity* (*Table III*).—When Landschütz tumour cells were incubated in 0·2 per cent trypsin at 37° C. for 60 minutes no effect on their subsequent growth in mice was detectable, i.e. trypsin alone had no apparent effect on their immunogenicity. However, subsequent treatment of these trypsinized cells with VCN for 30 minutes greatly increased their immunogenicity *in vivo*. VCN treated cells, however, lost their immunizing ability, after incubation in trypsin, suggesting that the antigenic sites exposed by VCN treatment are hydrolysed by the trypsin. VCN pretreated cells, trypsinized and subsequently incubated in VCN for a further 30 minutes remained non-immunogenic and produced tumour in all the recipient mice, implying that all available antigenic sites were exposed by the preliminary VCN treatment and then degraded by trypsin. Similarly, trypsinized cells rendered immunogenic by VCN treatment, when incubated in trypsin again lost their ability to induce immunity and killed all the mice.

*Ribonuclease and hyaluronidase* (*Table IV*)

Incubation in ribonuclease (RNAse) for 60 minutes produced a fall in the electrophoretic mobility of the tumour cells. On inoculation into mice, however, such cells produced death from malignant ascites in the same manner as untreated cells. Hyaluronidase treatment for 60 minutes produced no change in electro-

TABLE III.—*Effects of Trypsin and Various Sequential Trypsin/VCN Treatments on the Immunogenicity of the Tumour Cells*

| Treatment of cells | Temperature ° C. | Time (minutes) | Dye Exclusion per cent | Mice surviving at day 30 | Rechallenge with 5 × $10^4$ cells |
|---|---|---|---|---|---|
| VCN | 37 | 30 | 94 | 39/40 | 36/39 |
| Trypsin | 37 | 60 | 88 | 0/10 | — |
| VCN and Trypsin | 37<br>37 | 30<br>60 | 80 | 0/5 | — |
| Trypsin and VCN | 37<br>37 | 60<br>30 | 86 | 5/5 | 5/5 |
| Trypsin and VCN and Trypsin | 37<br>37<br>37 | 60<br>30<br>60 | 72 | 0/5 | — |
| VCN and Trypsin and VCN | 37<br>37<br>37 | 30<br>60<br>30 | 74 | 0/5 | — |

TABLE IV.—*Effects of Ribonuclease and Hyaluronidase on the Electrophoretic Mobility and on Subsequent Tumour Growth* in vivo

| Treatment of cells | Temperatue °C. | Time (minutes) | Dye exclusion per cent | Electrophoretic mobility μ/sec./v/cm. | Mice surviving at day 30 | Rechallenge with 5 × $10^4$ cells |
|---|---|---|---|---|---|---|
| HBSS | 37 | 30 | 97 | −1·317 | 0/10 | — |
| VCN | 37 | 30 | 94 | −0·690 | 39/40 | 36/39 |
| Ribonuclease | 37 | 60 | 89 | −1·026 | 0/10 | — |
| Hyaluronidase | 37 | 60 | 87 | −1·314 | 0/10 | — |

phoretic mobility and had no effect on the ability of the cells to develop and kill all the injected mice.

### DISCUSSION

Langley and Ambrose (1967) have studied the linkages of sialic acid on the surface of the Ehrlich ascites tumour and concluded that it mainly occupied the terminal positions on the oligosaccharide prosthetic groups of cell surface glycoproteins. Hydrolysis of these prosthetic groups revealed that the sialic acid was predominantly associated with N-acetyl D-galactosamine and that the molar ratio of these sugars approached unity. They therefore inferred that the prosthetic groups were disaccharides with sialic acid occupying the terminal non-reducing position (i.e. as N-acetyl neuraminosyl 2-? N-acetyl D galactosaminoyl-). They also detected the presence of N-glycolyl neuraminic acid on the same cells but indicated that its concentration was much less than sialic (N-acetyl neuraminic) acid. They also showed that the sialic acid content was associated with trypsin-labile proteins and not with cell wall lipids, thus confirming the observations of Gray (1963) who found only minute quantities of glycolipid in similar tumour cells.

FIG. 1.—A simplified glycoprotein prosthetic group (N-acetyl neuraminosyl 2–6 N-acetyl D galactosaminoyl-) showing the following features. (1)—N-acetyl neuraminic acid carboxyl group (2)—2–6 O-glycoside bond which is (3)—Site of action of neuraminidase. (4)—Probable ester glycosidic linkage to acidic amino acids in polypeptide chain. (After Gottschalk, 1960).

FIG. 2.—Simplified diagram of the possible relationship between a disaccharide prosthetic group (N-acetyl neuraminosyl 2–6 N-acetyl D galactosaminoyl) and an underlying trypsin sensitive site (tss) in the glycoprotein peptide chain. Access of trypsin to the tss (as shown on the right) is inhibited by the terminal sialic acid moiety. Host recognition of antigenic determinant activity involving the tss could be similarly inhibited by steric hindrance.

The action of neuraminidase is to catalyse the hydrolytic cleavage of O-glycoside bonds between the potential keto-groups of N-acylated neuraminic acids and adjacent sugar residues (Gottschalk, 1960). Treatment of the Landschütz ascites tumour with neuraminidase derived from *Vibrio cholerae* (VCN) results in greatly enhanced immunogenicity of the cells when they are subsequently inoculated into mice. Thus, a potent anti-tumour immunity can be induced in the recipient animals by the administration of VCN-treated cells. It was previously demonstrated (Currie and Bagshawe, 1967; Bagshawe and Currie, 1968) that VCN treatment did not modify the viability of the cells or their ability to grow in immunosuppressed animals. The admixture of VCN-treated (and washed) tumour cells with an equal number of untreated cells did not modify the ability of recipient mice to develop immunity. As the inoculation of these mice with treated and untreated cells together failed to induce tumour, then the immunizing properties of the VCN-treated cells must have been sufficient to allow the host to overcome the growth of the untreated cells and would imply that the enhanced immunogenicity of the treated cells need not be dependent upon modification of their growth potential. This is further supported by the observations of Kraemer (1966) who demonstrated that neuraminidase-treated cells will grow readily in tissue culture.

The masking of antigenicity must therefore be associated with the presence of N-acylated neuraminic acids in the surface glyocproteins of the tumour cells.

In the present studies there were dramatic differences between the effects of different species of neuraminidase on the immunogenicity of the Landschütz tumour. *Clostridium perfringens* neuraminidase (CPN) under incubation conditions identical to the VCN experiments (i.e. 30 minutes) failed to increase the immunogenicity of the tumour. However, incubation with CPN for 3 hours did apparently unmask the antigenicity of the cells. Inactivated influenza virus, rich in viral neuraminidase (FLU N) failed to modify the immunogenicity of the tumour cells even after incubation for long periods. An explanation for these important differences may be found by examining the site of action of neuraminidase on the substrate. Sialic acid may be linked to sugar residues by several types of O-glycoside bond. In most biological systems studied so far the commonest types of neuraminidase-sensitive bond are between the number 2 carbon atom of sialic acid and either the 3 or the 6 carbon atom of the adjacent aminosugar (Gottschalk, 1960). VCN is known to cleave both the 2–3 and the 2–6 bonds readily (Drzeniek, 1967), whereas myxovirus neuraminidases, including FLU N, only cleave O-glycoside bonds of the 2–3 type. Burton (1963) has examined the time course of hydrolysis of both 2–3 and 2–6 bonds by CPN and revealed dramatic differences. Incubation with CPN for 30 minutes in his system resulted in release of approximately 90 per cent of 2–3 bound sialic acid but only minute amounts of that linked by the 2–6 bond. However after 3 hours incubation the percentage of 2–6 bound sialic acid released rose to 95–100 per cent. Thus incubation of the Landschütz tumour cells in CPN for 30 minutes may only remove 2–3 bound sialic acid. Only after 3 hours' incubation is the 2–6 bond hydrolysed and the tumour cells rendered immunogenic. After CPN treatment of the cells for only 30 minutes however the cells were still susceptible to the unmasking effects of VCN, implying that the substrate sites were not blocked by inactive (or slow acting) CPN molecules. Hydrolytic cleavage of the disaccharide prosthetic group N-acetyl neuraminosyl 2–3 N-acetyl D galactosaminoyl alone, as would occur with FLU N, or CPN incubated for 30 minutes, is therefore insufficient to modify the immunogenicity of the tumour cells. Cleavage of N-acetyl neuraminosyl 2–6 N acetyl D galatosaminoyl- as well as the 2–3 prosthetic group must take place before the cells are capable of immunizing the recipient mice. It is not yet clear whether release of the 2–6 bound sialic acid alone is sufficient to unmask antigenic sites. Consequently it is not yet possible to explain the role played by the different types of O-glycoside bond in the immunogenicity of these tumour cells. A quantitative steric difference in the three dimensional positions of 2–3 and 2–6 bound sialic acid may account for this phenomenon. However, it is not inconceivable that a quantitative effect of the total concentration of sialic acid in the cell periphery, regardless of the type of bond, may be operating in the present system.

Gottschalk and Fazekas de St Groth (1960) have studied tryptic hydrolysis of a submaxillary sialomucin. The number of potential trypsin-sensitive sites cleaved was determined by assay of amide residues. They revealed that the number of sites hydrolysed by trypsin was greatly increased after prior neuraminidase treatment of the sialomucin. They therefore postulated that sialic acid inhibits tryptic hydrolysis by steric hindrance, i.e. the prosthetic group terminal sialic acid blocked underlying substrate sensitive sites from contact with the active centre of the trypsin molecule. It is of interest that the prosthetic groups they described were identical to those found by Langley and Ambrose (1967) on ascitic tumour cells. Furthermore the O-glycoside bond between the saccharides in this prosthetic group was of the 2–6 type. In the present experiments the amount of mucinous material released from tumour cells by trypsin was grossly increased by pre-treatment of the cells with VCN. This finding suggests that the labile sites on cell wall mucins were protected in a similar manner by neuraminidase sensitive sialic acid, i.e. steric hindrance.

The immunogenicity of VCN-treated Landschütz cells was abolished by trypsin treatment but pretreatment of the cells with trypsin did not abolish the effects of a subsequent VCN treatment, implying that the sensitive antigenic determinant sites were protected from the action of trypsin by sialic acid. Trypsin treatment, followed by VCN and then retrypsinization resulted in " non-immunogenic " cells and subsequent death of the recipient mice. Trypsinized VCN-pretreated cells could not, moreover, be rendered immunogenic by subsequent VCN treatments. This suggests that the preliminary VCN treatment exposed all available antigenic sites to the disruptive effects of trypsin. These results may be interpreted, in the light of Gottschalk and Fazekas de St. Groths' (1960) work on sialomucins, as implying that steric factors are involved in inhibiting access of the active centre of trypsin to sensitive sites in the antigenic areas. However Neurath and Schwert (1950) have pointed out that tryptic hydrolysis may be inhibited by the electrostatic effects of ionized carboxyl groups in the region of the sensitive site. The powerfully ionogenic carboxyl group of the terminal sialic acid could possibly provide such electrostatic effects. At the moment it is not possible to dissociate steric hindrance from the local effects of charged groups. The molecular basis of enzyme-substrate interactions is similar in many ways to the relationship between an antigen and its complementary receptor site. It seems feasible that mechanisms hindering the enzymatic degradation of a given antigenic site would also inhibit immunological interactions involved in the detection of antigens with the same antigenic determinant groups.

The nature of the antigen(s) exposed by the action of neuraminidase on the Landschütz tumour has not yet been explored. This tumour is a non-specific transplantable neoplasm which probably represents a malignant allograft. Therefore its antigenicity may well be related to the mouse histocompatibility antigen system. Nathensen and Davies (1966) have studied the chemical structure of solubilized H2 mouse isoantigens and believe them to be glycoproteins. The nature of the specific determinant groups on these glycoproteins is not yet clear but the hexosamine content of the prosthetic groups may be involved in such specificity. These preparations do contain sialic acid but neuraminidase treatment does not affect their antigenic activity (Davies, 1967). Thus the neuraminidase-sensitive sialic acids in these histocompatibility antigen preparations are probably not directly involved in the antigenic determinant site, although their underlying hexosamines may play a part. Therefore the elucidation of the structure of cell wall glycoproteins may have important implications for understanding the nature of antigenic determination and investigation of the role of sialic acid in thise glycoproteins may give some clues as to how the the expression of such antigenicity is moderated.

Neuraminidase treatment will expose antigens on the red cell. Saber, Drzeniek and Krüpe (1965) have shown that carbohydrate groups demonstrating ABH blood-group specificity may be unmasked by treatment of several species of erythrocytes with neuraminidase. Similarly, treatment of erythrocytes will expose Forssmann antigen activity under conditions where untreated cells show no such activity (Drzeniek *et al.*, 1966). Burnet and Anderson (1947) have also shown that treatment of red cells with R.D.E. (receptor destroying enzyme = neuraminidase) will bring about T-agglutination in many normal mammalian sera. They postulated that the enzyme had exposed a previously undetectable surface antigen on the red cells. It could be postulated that the effects of VCN on the Landschütz ascites tumour are also due to a form of T-agglutination. Our previous studies indicate that this is not so. The growth of VCN-treated cells in irradiated (Bagshawe and Currie, 1968) or hydrocortisonized mice (Currie and Bagshawe, 1968) and the lack of high titre agglutination of normal mouse sera for VCN-treated cells would indicate that factors other than T-agglutination must be involved.

Although the present studies have been confined mainly to sialic acid it is not inconceivable that other cell surface components may modify the expression of antigenicity by cells. Weiss (1968) has indicated that RNA may be an integral part of the cell periphery in several cell types. The effects of RNAse on the electrophoretic mobility of the Landschütz tumour suggest that RNA may well be present on the cell surface. However RNAse had no effect on the immunogenicity of this tumour. Hyaluronidase had no effect on either its electrophoretic mobility or its immunogenicity indicating the probable absence of hyaluronic acid from the glycocalyx of this particular tumour. However the chemical structure of the cell surface is undoubtedly very complex and further work is needed to evaluate the role of various other cell surface components in modifying the immunogenic properties of cells.

The relationship between the cell surface glycoproteins in the glycocalyx and such surface phenomena as contact inhibition, intercellular adhesiveness and antigenicity is not yet clear. Extensive studies of the action of neuraminidase on glycoprotein prosthetic groups on the cell surface may provide valuable information about the molecular structures responsible for moderating such cellular interactions and may give some insight into the " chemistry of antigenicity ".

SUMMARY

Experiments were performed to elucidate the role of sialic acid and its mode of linkage to the cell surface in inhibiting the detection of antigens on the Landschütz ascites tumour by the host's immunological mechanisms. The immunogenicity of this tumour was greatly enhanced after *in vitro* incubation in neuraminidase. The antigenic sites exposed by neuraminidase were destroyed by trypsin. However, prior treatment with trypsin did not affect the subsequent exposure of antigen(s) by neuraminidase. Various sequential enzyme treatments of the tumour cells with trypsin and/or neuraminidase suggest that the trypsin-sensitive components of the antigenic determinant groups are protected from the active centre of the trypsin molecule by neuraminidase-susceptible sialic acids. It is proposed that these antigenic sites may be protected from the host's immunocompetent cells in a similar manner.

Neuraminidases derived from different species of micro-organism display different effects on the immunogenicity of this tumour. *Vibrio cholerae* neuraminidase readily unmasked the antigenic sites on the tumour cells. The enzyme which comprises part of the influenza virion had no effect, whereas *Clostridium perfringens* neuraminidase only produced an effect after prolonged incubation. These enzymes have different substrate specificities and it is suggested that the enzymatic removal of cell wall sialic acid bound to hexosamines by a 2–3 O-glycosidic linkage is insufficient to unmask antigenic sites. Hydrolysis of the 2–6 bonds appears to be essential.

Ribonuclease and hyaluronidase treatment *in vitro* had no effect on the subsequent *in vivo* development of tumour cells. It was incidentally revealed that ribonucleic acid is probably present in the cell periphery of the Landschütz tumour cells.

It is suggested that the neuraminidase-sensitive terminal sialic acid moiety of cell surface glycoprotein prosthetic groups inhibits access to underlying antigenic determinant areas; this phenomenon probably depending on the type of O-glycoside bond between the sialic acid and underlying hexosamines. Although it is not yet possible to rule out the local inhibitory electrostatic effects of the carboxyl groups of sialic acid it seems probable that the blocking effects of this sialic acid are due to steric hindrance.

G.A.C. gratefully acknowledges a Research Fellowship from the Wellcome Trust. Studies in these laboratories are supported by the British Empire Cancer Campaign for Research, the Charing Cross Hospital Research Sub-Committee and the Post-Natal Chorionepithelioma Trust.

### REFERENCES

Abercrombie, M. and Ambrose, E. J.—(1962) *Cancer Res.*, **22**, 525.

Bagshawe, K. D. and Currie, G. A.—(1968) *Nature, Lond.*, in press.

Bangham, A. D., Flemans, R., Heard, D. H. and Seaman, G. V. G.—(1958) *Nature, Lond.*, **182**, 642.

Bennett, H. S.—(1963) *J. Histochem. Cytochem.*, **11**, 15.

BURNET, F. M. AND ANDERSON, S. G.—(1947) *Aust. J. exp. Biol. Med. Sci.*, **25**, 213.
BURTON, R. M.—(1963) *J. Neurochem.*, **10**, 503.
CURRIE, G. A.—(1967) *Lancet*, ii, 1336.
CURRIE, G. A. AND BAGSHAWE, K. D.—(1967) *Lancet*, i, 708.—(1968) *Br. J. Cancer*, **22**, p. 588.
DAVIES, D. A. L.—(1967) ' Cross reacting antigens and neoantigens '. Edited by J. Trentin. Baltimore (Williams and Wilkins).
DRZENIEK, R.—(1967) *Biochem. biophys. Res. Commun.* **26**, 631.
DRZENIEK, R., SABER, M. S. AND ROTT, R.—(1966) *Z. Naturf.*, **21**, 254.
FAWCETT, D. W.—(1964) ' Modern developments in electron microscopy '. Edited by B. M. Siegel. New York (Academic Press).
GASIC, G. AND BEYDAK, T.—(1961) ' Biological interactions in normal and neoplastic growth'. Edited by M. J. Brennan and W. L. Simpson. London (Churchill), p. 709.
GOTTSCHALK, A.—(1960) 'The chemistry and biology of the sialic acids and related substances '. London (Cambridge University Press).
GOTTSCHALK, A. AND FAZEKAS DE ST. GROTH, S.—(1960) *Biochim. biophys. Acta*, **43**, 513.
GRAY, G. M.—(1963) *Biochem. J.*, **86**, 350.
KRAEMER, P. M.—(1966) *J. cell. comp. Physiol.*, **68**, 85.
LANGLEY, O. K. AND AMBROSE, E. J.—(1967) *Biochem. J.*, **102**, 367.
LINDENMANN, J. AND KLEIN, P. A.—(1967) ' Immunological aspects of viral oncolysis '. New York (Springer-Verlag).
NATHENSEN, S. G. AND DAVIES, D. A. L.—(1966) *Ann. N.Y. Acad. Sci.*, **129**, 6.
NEURATH, H. AND SCHWERT, G. W.—(1950) *Chem. Rev.*, **46**, 69.
RAMBOURG, A. AND LEBLOND, C. P.—(1967) *J. Cell Biol.*, **32**, 27.
SABER, M. S., DRZENIEK, R. AND KRÜPE, M.—(1965) *Z. Naturf.*, **20**, 965.
SANFORD, B. H.—(1967) *Transplantation*, **5**, 1273.
WEISS, L.—(1958) *Exp. Cell. Res.*, **14**, 80—(1968) *J. theor. Biol.*, **18**, 9